# Laser Scribed Graphene Biosensor for Detection of Biogenic Amines in Food Samples Using Locally Sourced Materials

**DOI:** 10.3390/bios8020042

**Published:** 2018-04-24

**Authors:** Diana C. Vanegas, Laksmi Patiño, Connie Mendez, Daniela Alves de Oliveira, Alba M. Torres, Carmen L. Gomes, Eric S. McLamore

**Affiliations:** 1 Department of Food Engineering, Universidad del Valle, Cali 760032, Colombia; laksmi.patino@correounivalle.edu.co (L.P.); connie.mendez@correounivalle.edu.co (C.M.); 2 Department of Biological and Agricultural Engineering, Texas A&M University, College Station, TX 77843, USA; daoliveira@tamu.edu; 3 Department of Biology, Universidad del Valle, Cali 760032, Colombia; albamarina.torres@gmail.com; 4 Department of Mechanical Engineering, Iowa State University, Ames, IA 50011, USA; carmen@iastate.edu; 5 Department of Agricultural and Biological Engineering, Institute of Food and Agricultural Sciences, University of Florida, Gainesville, FL 32611, USA; emclamore@ufl.edu

**Keywords:** biogenic amines, laser scribed graphene, diamine oxidase, disposable sensor, food quality, risk assessment, planetary health

## Abstract

In foods, high levels of biogenic amines (BA) are the result of microbial metabolism that could be affected by temperatures and storage conditions. Thus, the level of BA is commonly used as an indicator of food safety and quality. This manuscript outlines the development of laser scribed graphene electrodes, with locally sourced materials, for reagent-free food safety biosensing. To fabricate the biosensors, the graphene surface was functionalized with copper microparticles and diamine oxidase, purchased from a local supermarket; and then compared to biosensors fabricated with analytical grade materials. The amperometric biosensor exhibits good electrochemical performance, with an average histamine sensitivity of 23.3 µA/mM, a lower detection limit of 11.6 µM, and a response time of 7.3 s, showing similar performance to biosensors constructed from analytical grade materials. We demonstrated the application of the biosensor by testing total BA concentration in fish paste samples subjected to fermentation with lactic acid bacteria. Biogenic amines concentrations prior to lactic acid fermentation were below the detection limit of the biosensor, while concentration after fermentation was 19.24 ± 8.21 mg histamine/kg, confirming that the sensor was selective in a complex food matrix. The low-cost, rapid, and accurate device is a promising tool for biogenic amine estimation in food samples, particularly in situations where standard laboratory techniques are unavailable, or are cost prohibitive. This biosensor can be used for screening food samples, potentially limiting food waste, while reducing chances of foodborne outbreaks.

## 1. Introduction

Food waste is a major problem across the planetary health spectrum; it is estimated that 1.3 billion tons of food is wasted each year. This equates to a global cost of 1 trillion dollars in food loss, and a considerable waste of energy and water resources [1]. While most food waste occurs in developed countries, developing countries and inaccessible rural regions bear most of the global health burden for foodborne diseases [2,3,4]. In fact, according to the World Health Organization [5], the global burden of foodborne disease is at least 33 million disability adjusted life years (DALYs), which is comparable to that of HIV/AIDS, malaria or tuberculosis. While pathogenic bacteria are the primary cause of the noted DALYs in developing regions, small molecules and toxins are attributed to most acute toxicity cases, and are a particularly dangerous problem for those with compromised immune systems. Thus, there is a critical need for the development of low-cost, rapid sensors, that can be used to accurately monitor small molecules and toxins that serve as food quality/safety indexes; such a development would provide a useful insight into the real quality and preservation conditions of food products, and as a consequence, enable risk-reduction of food waste and foodborne illnesses, particularly in developing regions that do not have easy access to analytical laboratories [6,7,8].

Biogenic amines (BA) are basic nitrogenous compounds of global toxicological significance, which are directly linked to food safety and also food waste and quality—and consequently human health. Presence of high levels of BA in food can result in food poisoning, while low levels may lead to food intolerance [9]. BA in food can originate from endogenous enzymatic activity, or by microbial metabolism, which leads to either decarboxylation of amino acids, or amination of aldehydes and ketones [10]. The properties of individual BA (e.g., histamine, tyramine, cadaverine) vary depending on the amino acid precursor (e.g., histidine, tyrosine, lysine) and chemical structure (aliphatic, aromatic, or heterocyclic). The total BA in any given food product is a function of the particular biochemical composition and the type and amount of microorganism present [11]. For instance, fermented foods such as cheese, wine, sausage, and pickled vegetables, which use dense communities of lactic acid bacteria to drive the fermentation, can potentially contain large concentrations of histamine, cadaverine, tyramine, and/or putrescine [12]. Fermented fish products are particularly susceptible to high levels of BA due to the combination of high microbial loads and a high content of amino acid precursors. Since accumulation of histamine, putrescine, cadaverine, tyramine, trimethylamine, and dimethylamine can be correlated to microbial contamination, the total concentration of BA is commonly used to estimate quality and safety indices, as well as the overall shelf-life in fish, fish products, and shellfish [13].

Maximum thresholds of BA vary, depending on particular regulations of each country. In the European Union, (EC) 2073/2005 lays down food safety limits for histamine between 100–200 mg/kg in “fishery products from fish species associated with a high amount of histidine”, and between 200–400 mg/kg for “fishery products which have undergone enzyme maturation treatment in brine, manufactured from fish species associated with a high amount of histidine” [14]. In the United States, the FDA considers food as spoiled if the histamine level reaches 500 mg/kg [15]. In Canada, Switzerland and Brazil the maximum permissible limit of histamine in fish and fishery products is 100 mg/kg. The Australian and New Zealand Food Standards Code states that the level of histamine in fish or fish products must not exceed 200 mg/kg [16]. In Colombia, food safety regulations also allow histamine levels up to 200 mg/kg [17]. Standardized laboratory techniques for detecting BA in food samples include capillary electrophoresis and chromatographic methods, such as thin layer chromatography, high-performance liquid chromatography, and gas chromatography. Although highly accurate, these methods are expensive and time consuming, requiring several sample pretreatments with external reagents, as well as multiple derivatization steps [18]. Unfortunately, a large number of fermented foods and fish markets are located in developing countries or marine areas, where access to laboratory techniques and resources are limited due to the lack of certified facilities, as well as the high analytical cost. Thus, detection of BA is not performed with sufficient frequency in most food processing facilities, leading to a significant number of food-related disease outbreaks every year [19,20,21]. New, reagent-free technologies with fast analysis time, relevant detection limits, low-cost, and simple acquisition hardware are clearly needed, in order to enable the food industry to perform more accurate risk assessments of BA contamination. Rapid analysis informs managers, allowing them to enact timely and appropriate quality and safety measures to limit consumer exposure to these hazardous compounds. To achieve this, there are many biorecognition-transduction approaches that are viable for BA sensing, as reviewed by Vanegas et al. [22]. One of the most popular devices for BA uses diamine oxidase (DAO) as the biorecognition structure to selectively oxidize BA in electrochemical biosensing.

While many electrochemical sensor platforms exist for creating DAO biosensors, nanocarbon-based biosensors (e.g., graphene, carbon nanotube, etc.) are among the most promising. Mask-free paper-based devices, such as the conductive graphene paper by Burrs et al. [23], are useful, but require significant effort/time to fabricate, and batch-to-batch variation is too high for screening large batches of food product. Zhang et al. [24] developed a lithography-free technique for creating laser-reduced graphene oxide (GO) films for electronic applications. This was later expanded on by Lin et al. [25], to facilitate direct laser writing of flexible graphene circuits without need for the deposition of a GO slurry prior to laser treatment. This transformative advancement allows any lab to directly write conductive circuits on commercial polymers (polyimide) with a single laser setup. The technique, known as laser scribed graphene (LSG), converts sp^3^ carbon in the polyimide film to sp^2^ carbon, which is the allotrope found in graphene [26,27,28]. Recent studies of LSG are focused on understanding material properties for improving engineering of this new material on various substrates [29].

**Figure 1:**
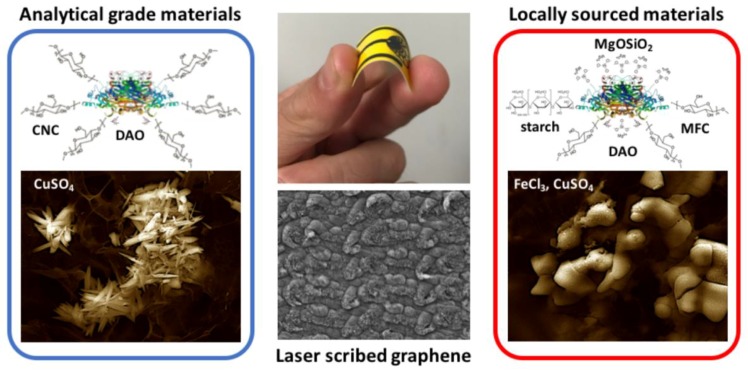
A low-cost, disposable nanobiosensor was developed for measuring biogenic amines in food using locally sourced materials, and was compared to sensors fabricated with analytical grade materials.

Direct synthesis of LSG on polyimide films significantly reduces cost and complexity compared to solution-prepared GO films, thus facilitating the development of disposable, low-cost sensors, such as the glucose sensors by Tehrani and Bavarian [30]. This exciting new technique has been further expanded upon for measuring organic acids such as dopamine [31] and thrombin [32]. Fabrication of LSG electrodes is reproducible by any sensor laboratory, thus opening the potential for manufacturing sensors that can be used in challenging environments, such as rural or economically limited areas. To make this a reality, studies are needed to expand the number of analytes amenable to LSG electrochemistry using low-cost and locally sourced materials. Thus, this study focuses on development of LSG biosensors for the detection of BA levels in food samples. To allow future applications in food safety monitoring by small-scale facilities in developing countries, biosensors are demonstrated using locally sourced materials to both metallize and biofunctionalize the surface for BA detection (Figure 1). The general sensor performance characteristics (limit of detection, response time, sensitivity, and selectivity) were compared to LSG electrodes fabricated with analytical grade metals and high purity enzymes, to validate the approach. Finally, we apply the biosensor developed with locally available materials for analysis of biogenic amines in fermented fish under conditions which represent economically challenged regions.

## 2. Materials and Methods

### 2.1. Materials and Reagents

Potassium nitrate (KNO_3_), hydrogen peroxide 35% (*w*/*v*), and histamine 97% (*w*/*w*) were purchased from Acros organics (Springfield Township, NJ, USA). Potassium ferrocyanide trihydrate (K_3_Fe(CN)_6_) was acquired from HiMedia Laboratories (Mumbai, India). Phosphate buffer saline (PBS 1X) was prepared with sodium phosphate dibasic (NaHPO_4_), potassium phosphate (KH_2_PO_4_), potassium chloride (KCl), and sodium chloride (NaCl), obtained from Chem Center (La Jolla, CA, USA). Copper sulfate (23.5 wt %, as fertilizer) was acquired from an agricultural supply store (Campofert S.A.S., Yumbo, Colombia).

Purified diamine oxidase (DAO) from porcine kidney was obtained from Sigma Aldrich (St. Louis, MO, USA). DAO (7 wt % as dietary supplement) was obtained from Swanson Health Products, Inc. (Fargo, ND, USA). The formulation contained microcrystalline cellulose, sucrose, ascorbic acid, rice starch, hydrated magnesium silicate, corn starch, carboxymethylcellulose, titanium dioxide, and glycerol (proprietary concentrations). Kapton (polyimide) tape, of approximately 30.4 μm film thickness and 50 mm width, was obtained from McMaster-Carr Co. (Elmhurst, IL, USA), silver/silver chloride ink for screen printing was acquired from DuPont (Wilmington, DE, USA). Nine volt batteries were acquired from a local hardware store.

### 2.2. Electrode Fabrication

LSG electrodes were fabricated based on a technique reported by Teharani and Bavarian [30]. Briefly, a three-electrode system (working, reference, and auxiliary electrodes) was designed using Microsoft paint (Microsoft Corp., Redmond, WA, USA). Kapton tape was applied to photo paper (the polyimide was placed on the emulsion side of the paper), and the composite was fixed in the base of a UV laser engraver (λ = 405 nm; HTPOW 1000 mW Mini USB Laser Engraver by HTPOW Laser Limited, Shenzhen, China) with two-dimensional stepper motors. A laser pulse rate of 21 ms (laser energy density of 1.7 J cm^−2^), beam size of 1.3 mm, and laser-to-substrate distance of 13 cm, was used for all sensor fabrication. Next, silver/silver chloride ink was applied to the reference electrode, as well as on the bonding pad of all three electrodes to prevent electrode fracture from repeated use. Finally, a passivation layer of fast drying nitrocellulose lacquer (approximately 200 µm thick) was applied between the electrodes and the connectors to insulate the non-active features. Supplemental Figure S1 depicts the fabrication process of the sensor platform.

### 2.3. Copper Electrodeposition

Copper nanocubes were electroplated on the surface of the working electrode, based on Tehrani and Bavarian [30]. The working electrode was connected to the anode of a 9 V battery, and copper was used as the cathode during electrodeposition at 25 °C for various durations, as noted (see below for details). Two different plating solutions were compared, with one solution representing locally sourced materials, and the other representing standard, analytical grade materials. For the sensors fabricated with locally sourced materials, a commercial fertilizer containing CuSO_4_ (250 mM) was used with a copper coin as cathode. For electrodeposition using analytical grade chemicals, a solution of 250 mM CuSO_4_ and 2.5 mM Na_2_SO_4_ was used with a copper mesh (diameter 0.5 mm) as the cathode. Prior to use, the copper mesh was polished for 30 s at 9 V in a solution of 25% (*v*/*v*) ethanol and 25% (*v*/*v*) phosphoric acid. After deposition of metal, electrodes were rinsed with DI water, to remove any excess materials from the surface, and allowed to dry at room temperature.

### 2.4. Material Characterization

In order to verify the particle size and topography of the working electrode, SEM images were obtained using a JEOL 5600 LV, with an accelerating voltage of 12–20 kV. Electron dispersive X-ray spectroscopy (EDS) was carried out using a solid state EDAX silicon drift detector (20,000×) on a SEM 6400. A Horiba Aramis Raman spectrometer with a 532-nm laser source was used to obtain Raman spectra. The Raman spectrometer settings for all samples also included a confocal hole of 100 nm with a 1–3% transmission filter (range from 400 to 3000), at 20 s per scan and 1× ad hoc averaging.

### 2.5. Sensor Biofunctionalization

In order to provide selectivity toward biogenic amines, LSG electrodes were biofunctionalized with DAO, based on methods modified from Burrs et al. [33]. DAO was encapsulated with cellulose (either microfibrilated or nanocrystalline), and then cross-linked. An enzyme solution (0.72 mg/mL, approximately 25,000 histamine degrading units) was prepared by dissolving lyophilized DAO, or the contents of one DAO capsule, in 5 mL of PBS buffer (pH 7.4), as noted. Next, the solution was stirred for 30 min at 25 °C, and 5 µL was drop-cast on the surface of the LSG working electrode and dried at 25 °C for one minute. Next, 5 µL of glutaraldehyde (2 wt %) was drop-cast on top of the electrode to cross-link the enzyme and prevent protein leaching. Finally, biosensors were dried for 1 h at 4 °C prior to use. The operating mechanism of the obtained biosensor is based on the catalytic reaction shown in Equation (1).

(1) $$R-{\mathrm{CH}}_{2}-{\mathrm{NH}}_{2}+H_{2}O+O_{2}\overset{\mathrm{DAO}}{\to }R-\mathrm{CHO}+{\mathrm{NH}}_{3}+H_{2}O_{2}$$

$$H_{2}O_{2}\overset{500\;\mathrm{mV}}{\to }2H^{+}+O_{2}+2e^{-}$$

DAO enzyme catalyzes the deamination of primary amines, diamines, and substituted amines via oxidation, to generate aldehyde, ammonia, and hydrogen peroxide [34]. Hydrogen peroxide is then readily decomposed at a working electrode polarized at +500 mV, producing an oxidative current. This electrical response can be further correlated to the concentration of BA in the electrochemical cell via calibration plots.

### 2.6. Electrochemical Characterization

A modular potentiostat (EA163, eDAQ, Denistone East, Australia) was used to perform electrochemical characterization. Cyclic voltammetry was performed in a solution of 4 mM Fe(CN)_6_/1 M KNO_3_ at an initial potential of −1 V and switching potential of 1 V, with incremental scan rates of 50, 100, 150, and 200 m-s^−1^. The Randles–Sevcik theorem was used to determine the electroactive surface area of the working electrode, as shown in Equation (2):

(2) $$i_{p}\;=\;\left(2.69\times 10^{5}\right)n^{1.5}D^{0.5}CAv^{0.5}$$

where: *i_p_* (ampere) is the oxidation peak acquired from the cyclic voltammogram, *n* is the number of transferred electrons in the oxidation-reduction reaction, *D* is the coefficient of diffusion (cm^2^-s^−1^), *C* is the concentration of redox probe (mM), *A* is the electroactive surface area of the electrode (cm^2^), and *v* is the potential scan rate (mV-s^−1^).

The electroactive surface area (*A*) was calculated using the slope of the Cottrell plot (*i_p_* versus *v*^0.5^) and the known properties *n*, *D*, and *C* of the working solution, as described in our previous work [33,35]. The heterogenous electron transfer (HET) constant was calculated based on the method by Nicholson [36], using Equations (3) and (4), below. The value of the charge transfer parameter (*ψ*) is inversely proportional to the peak anodic/cathodic potential separation (∆E_p_), and can be calculated from various plots, as described by Siraj et al. [37], depending on whether the value of ∆E_p_ is near-Nernstian (59.16 mV/log C), or super-Nernstian.

(3) $$\psi \;=\;\frac{\gamma^{\alpha }*k_{s}}{{\left(\pi *a*D_{o}\right)}^{0.5}}$$

(4) $$a\;=\;\frac{nFv}{RT}$$

where: *ψ* is the dimensionless charge transfer parameter, *γ* is the dimensionless quantity *D_O_*/*D_R_*, *D_O_* is the diffusion coefficient for the oxidation state of redox probe (cm^2^-s^−1^), *D_R_* is the diffusion coefficient for the reduction state of redox probe (cm^2^-s^−1^), *α* is the transfer coefficient (0.5), *k_s_* is the HET constant (cm-s^−1^), *F* is the Faraday constant (96,485 C-mol^−1^), R is the universal gas constant, and *T* is the temperature (*K*).

DC potential chronoamperometry (DCPA) was performed using a working potential of +500 mV versus Ag/AgCl reference electrode and sampling rate of 1 kHz in PBS (pH 7.4) (Chart software, eDAQ Pty Ltd., Denistone East, Australia). After 60 min of polarization, the current output was obtained at a +500 mV constant potential, while successively injecting histamine in the working solution at 1 min intervals in order for the electrical signal to reach steady state. DCPA curves were used to evaluate the performance of the biosensor with regard to response time, sensitivity, and lower limit of detection.

Response time (t_95_) was calculated by taking the average of the 95% steady-state response time of three successive step changes in concentration over the linear range tested (between 0 and 1.75 mM histamine). Non-linear regression over single step changes in concentration (exponential rise to maximum) was performed using SigmaPlot 12.0, in order to obtain the steady-state response. The slope of the linear portion of the DCPA curves was used to obtain the sensitivity. The 3σ method was used to calculate the lower limit of detection (LOD), according to published methods [35,38].

### 2.7. Analysis of Fermented Fish

Proof of concept experiments were carried out by testing BA in non-fermented and fermented fish paste. Samples of a tilapia (*Oreochromis* spp.) paste were obtained from the applied microbiology laboratory at Universidad del Valle, Cali, Colombia. Samples of fish paste were taken prior to and after inoculation with a cocktail of lactic acid bacteria (*Lactobacillus alimentarius* and *Carnobacterium piscicola;* both strains are no BA-producing bacteria), used for a 6-day batch fermentation at 25 ± 5 °C. Samples were prepared by mixing 1 g of fish paste (non-fermented or fermented, as noted) with 9 mL of peptonized water. This mixture was vortex-agitated for 30 min to obtain a homogenized suspension, and then centrifuged at 4000 rpm for 15 min. After centrifugation, the supernatant was decanted and stored in glass vials at −10 °C until analyzed. Total BA concentration was determined via amperometric experiments in PBS buffer (pH 7.4) after calibration with histamine. The biosensor was first polarized at +500 mV for 1 h, and then 5 µL aliquots of the obtained supernatant were successively injected into the electrochemical cell and the current response was recorded. BA concentration was determined using the histamine calibration curves, the total BA was then estimated by calculating the normalized peak area for each injection. For calculating the normalized peak area, the peak current (*i_p_*) was calculated for each injection and normalized relative the baseline current (*i_o_*). The integral was then calculated, as shown in Equation (5), where *dt* is the total time step for each injection (16 min). Results were reported as mg-histamine/kg-fish paste.

(5) $$Norm.\;peak\;area\;=\;\int \left(i_{p}-i_{o}\right)*dt$$

### 2.8. Statistical Analysis

Measurements were made at least in triplicate as independent experiments, based on a completely randomized design for all analysis, and results were expressed as mean ± standard deviation. Statistical analysis was performed using SigmaPlot software (version 12.0), and variables were tested for significance by one-way analysis of variance (ANOVA), with Tukey test for significantly different means (*p* < 0.05).

## 3. Results and Discussion

### 3.1. LSG Morphology

**Figure 2:**
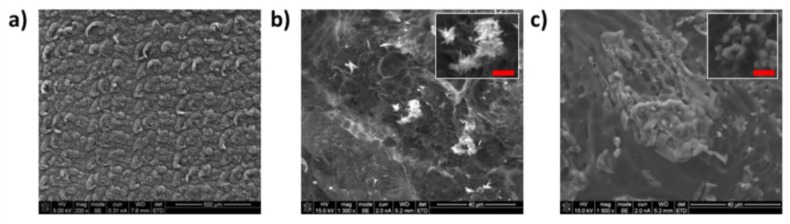
Morphological characterization of LSG electrodes. (**a**) Low magnification SEM of LSG, prior to metallization with copper; (**b**) high resolution SEM of electrode metallized with CuSO_4_; inset shows magnification of a copper microparticle (scale bar represents 5 µm); (**c**) high resolution SEM of electrode metallized with locally sourced material; inset shows magnification of a copper microparticle (scale bar represents 5 µm).

Low magnification SEM of the LSG (Figure 2a) shows a “stitched” like pattern, which is a result of the laser rastering across the polyimide surface during pulsed irradiation at 1 W. In preliminary studies, laser energy density lower than 1 J/cm^2^ or above 3 J/cm^2^ resulted in LSG that was not conductive, due to poor connectivity or large cracks respectively. The burr-like LSG microstructure has a high surface roughness, which provides a high surface area for metallization and subsequent protein immobilization. The average thickness of the LSG material was 63 ± 6 µm, and the paper substrate was approximately 350 µm thick (see Supplemental Figure S2). High resolution SEM images after metallization with analytical grade CuSO_4_ (Figure 2b) show exposed edge/plane porous graphitized structures that are decorated by nano and micron scale copper urchin-like structures. The edges of graphitized carbon in the porous 3D structure are known to have high chemical reactivity due to breaking of the local π conjugation [39,40]. Urchin-like nanostructures formed from CuSO_4_ electrodeposition ranged from 25 to 300 nm in diameter (*n* = 50 structures analyzed), with a mean diameter of 110 ± 71 nm. Fewer than 1.0% of the mesostructures were larger than 500 nm, although some of the aggregates were as large as 4 µm. When the locally sourced copper solution was used for metallizing LSG, the morphology of the surface changed considerably (Figure 2c). Rod-like structures were observed (500–2000 nm in diameter), some of which formed large meso-tube forms of up to 7 µm in length. The edge and plane features of the underlying LSG were no longer visible, and the material covered the entire surface. The mesotube structures were covered in amorphous microstructures that were composed of 1–2 µm nodules (see inset in Figure 2c). Formation of copper nanotube morphologies is possible, as Prucek et al. [41] reported, in the formation of nanoscale (18 nm diameter) copper tubes via chaining of copper nanocubes, but the structures shown in Figure 2c are much larger and less organized than those reported by others. It is unlikely that these structures are chained nanocubes or other copper structures, due to the lack of either clearly delineated nanostructures or apparent fractal features. Raman spectroscopy, EDS and XPS were used to determine the composition of the underlying structures for the morphologies shown in Figure 2b,c.

### 3.2. Material Analysis

**Figure 3:**
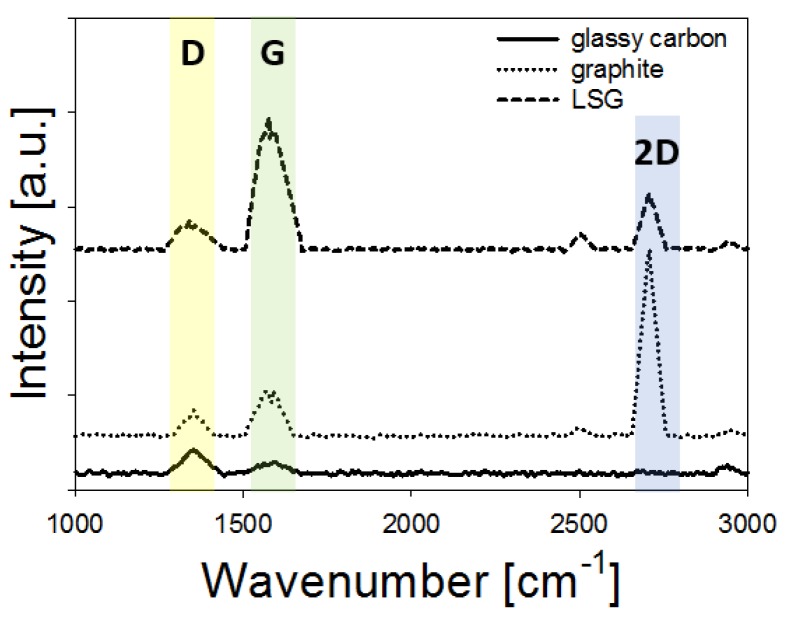
Raman spectra for glassy carbon electrode, graphite, and LSG. Characteristic peaks are apparent for D, G and 2D bands for the LSG, which is associated with graphene.

Raman spectra of the LSG (Figure 3) showed characteristic graphitic peaks (D at 1353 cm^−1^, G at 1586 cm^−1^, and 2D at 2704 cm^−1^) that are similar to published literature [30,31,32]. Room temperature laser-induced pyrolysis of the aromatic groups in polyimide (aromatic dianhydride and diamine) led to subsequent carbonization (transition from sp^3^ carbon to sp^2^ carbon), as shown by others [42,43]. Based on the approximation by Das et al. [44], the I_G_/I_2D_ ratio (0.60) obtained for the LSG indicates that the native LSG structure is composed of multilayer porous graphene. Although not studied in detail here, Wu et al. [29] showed that laser energy density is the most important factor for graphitization of the polymer, and care should be taken to control the laser energy density. We determined that a laser energy density of approximately 1.7 J/cm^2^ was optimal based on preliminary testing.

**Figure 4:**
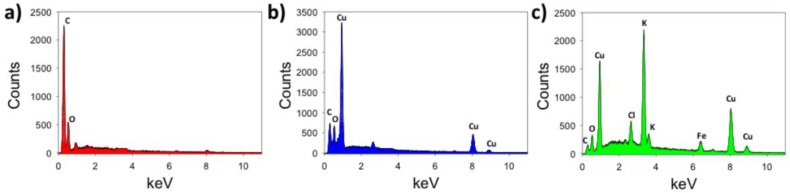
EDS spectra of LSG before and after metallization. (**a**) LSG after graphitization at 1.7 J/cm^2^, showing formation of graphitized carbon; (**b**) EDS spectra of the urchin structure shown in the inset of Figure 2b; (**c**) EDS spectra of nodules along large mesotube structures shown in the inset of Figure 2c.

EDS spectra of the prepared LSG (Figure 4a) showed peaks associated with carbon (0.28 keV) and oxygen (0.54 keV) in all analyzed samples. After electroplating with analytical grade CuSO_4_, clear copper peaks at 0.92 keV (Lα) and 8.1 keV (Kα) were measured in 93% of the spectra analyzed (*n* = 15 samples analyzed). The EDS spectra in Figure 4b are taken from the nanourchin structure, shown in Figure 2b. The carbon peak was considerably lower after metallization for all samples, which confirms that LSG was coated with the copper. After metallization with the locally sourced copper (Figure 4c), peaks associated with potassium (3.3 keV) and chloride (2.60 keV) were also apparent. In addition, a small peak at 6.97 keV associated with iron Kα was detected. These peaks were expected, as the local copper source contained a mixture of copper salts, iron chloride, and other trace elements, as noted by the manufacturer. The EDS spectra in Figure 4c were taken from the mesotube structure, pictured in Figure 2c inset, which shows that these structures did indeed contain copper, but the hybrid material was an alloy (iron, copper) that also contained potassium and chloride impurities.

XPS data (see Supplemental Figure S3) confirm that laser-induced pyrolysis of polyimide occurred for the as prepared LSG working electrode. The C1s peak (284.5 eV, 93.2% carbon mass) for LSG was significantly higher in carbon content than native polyimide, and the nitrogen and oxygen peaks were lower due to volatilization of aromatics, as indicated by the relatively low N1s peak (401.1 eV, 2.06% nitrogen mass) and O1s peak (533.00 eV, 4.32% oxygen mass). As a comparison, native polyimide is composed of 70.5% C, 7.0% N, and 22.5% O [45]. After metallization with copper, the Cu2p peak was significantly higher for both types of Cu-plated electrodes, and the low intensity N1s (401.1 eV; 2.47% N) and O1s peaks (533.00 eV; 3.18% O) were significantly lower than the LSG, which is similar to copper nanocubes formed on LSG as described by Tehrani and Bavarian [30]. Next, electrochemical properties were investigated for each of these LSG electrodes.

### 3.3. Electrochemical Characterization

**Figure 5:**
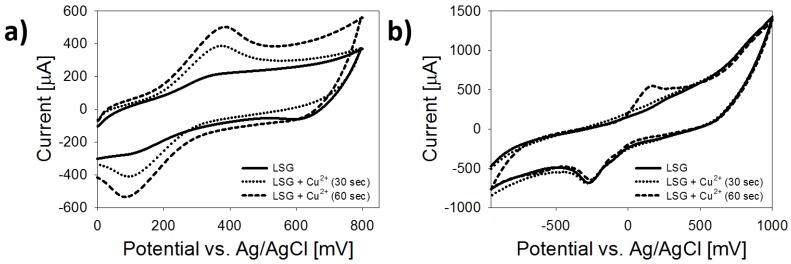
Representative CV at 100 mV/s for LSG electrodes after different Cu^2+^ electrodepositions using CuSO_4_ and locally sourced copper. (**a**) CV for LSG after metallization with CuSO_4_; (**b**) CV for LSG after metallization with locally sourced copper.

Figure 4 shows the effect of copper deposition time on the electrochemical response using both types of copper to metallize LSG. Cyclic voltammograms were acquired in 4 mM K_4_FeCN_6_ with 1 M KNO_3_, at a scan rate of 100 mV/s (room temperature) versus Ag/AgCl reference electrodes. After Cu^2+^ electrodeposition using CuSO_4_, well-defined oxidation and reduction peaks were observed; peak current (*i_p_*) was approximately 390 μA for 30 s deposition and 500 μA for 60 s (Figure 5a). After 30 s of deposition for locally sourced copper (Figure 5b), oxidation and reduction peaks at 100 mV/s were 97 and 112 μA respectively. Sharp peaks with ∆E_p_ > 300 mV were observed, indicating slow electron transfer kinetics relative to Figure 5a. After 60 s of deposition, the oxidative peak current (25 μA) and reduction peak (35 μA) increased significantly, but the potential separation did not change significantly. Electroactive surface area and heterogenous electron transfer constants were determined for each type of LSG electrode, to understand the effect(s) of copper metallization on sensor performance.

**Figure 6:**
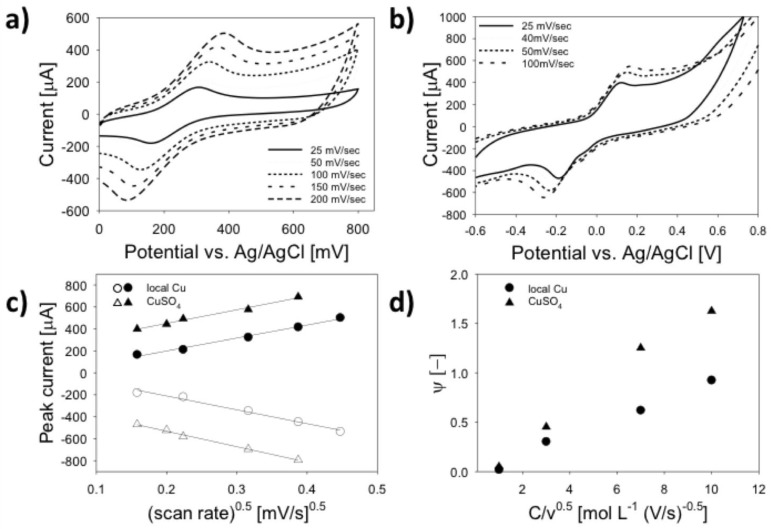
Representative CVs at various scan rates for LSG metallized with two types of copper source material. (**a**) CuSO_4_-plated LSG electrodes display reversible redox peaks; (**b**) LSG electrodes plated with locally sourced copper display quasi-reversible redox peaks below 100 mV/s, but no oxidation peaks are discernable above 100 mV/s (data not shown); (**c**) cottrell plots used to calculate ESA and HET constant were linear (*R*^2^ > 0.98) between 25 and 200 mV/s for CuSO_4_-plated LSG electrodes; (**d**) HET plots used to calculate k^0^ were linear between 25 and 100 mV/s (*R*^2^ > 0.97) for locally sourced copper-plated LSG electrodes. The geometric surface area of the LSG working electrode was 0.12 cm^2^.

Figure 6 depicts CVs obtained at various scan rates for Cu-plated LSG using each of the two copper sources. For all scan rates tested, LSG electrodes plated with analytical grade CuSO_4_ displayed well-defined redox peaks (Figure 6a). Linear Cottrell plots and HET plots (Figure 6b) indicate diffusion-limited charge transfer with an average electroactive surface area (ESA) of 0.46 ± 0.06 cm^2^ and HET constant (k^0^) of 0.003 ± 0.001. The HET value is similar to Fenzl et al. [32], which exceeds the k^0^ of graphite. This confirms the results in Figure 3, which indicated that the LSG structure was composed of multilayer porous graphene based on a I_G_/I_2D_ ratio of 0.60. When electroplated with analytical grade CuSO_4_, the average ESA and HET constant were approximately 20–25% higher, which is attributed to the high surface area nano-urchin structure that decorated the LSG surface, as well as the large number of exposed graphene edges shown in Figure 2b. Electrodes metallized with locally sourced copper (Figure 6b) produced well-defined oxidation/reduction peaks at or below 100 mV/s; however, as the scan rate increased, the oxidation peak was not discernable, although the reduction peak was distinct. This is due to the relatively slow chemical reaction during the initial CV sweep (see Table 1 and discussion for details). In this case, the ESA was calculated based on the reduction peaks to capture electrokinetic effects above 100 mV/s. It is also noteworthy that the peak separation (∆E_p_) for LSG fabricated with locally sourced copper was larger than 300 mV, which is beyond the limit for use of the dimensionless transformation by Nicholson [36]; consequently, the method proposed by Lavagnini et al. [46] was used for all calculations. The ESA and HET only increased by 8–11% when using the locally sourced copper, which is significantly lower than LSG metallized with analytical grade CuSO_4_. Figure 6c,d show the Cottrell plot and HET plot used to calculate ESA and k^0^ respectively. Note that k^0^ was only calculated for scan rates between 10 and 100 mV/s, since oxidation peaks were not discernable above 100 mV/s.

The following section shows sensor biofunctionalization and calibration for LSG electrodes after 30 s of metal deposition, using various types of enzyme (analytical grade and locally sourced).

### 3.4. Sensor Biofunctionalization and Calibration

Two different protein sources were used to fabricate copper-coated LSG electrodes metallized with locally sourced copper, namely, analytical grade DAO (Sigma Aldrich) and a DAO capsule purchased from a health food store, designed for human consumption. For fabricating biosensors with laboratory grade lyophilized powder, nanocrystalline cellulose hydrogel (CNC; USDA Forest Products Laboratory) was used as an encapsulant based on the methods described by Burrs et al. [33]. These copper-coated LSG biosensors are referred to as CNC-DAO throughout. For sensors prepared with the locally purchased protein powder, the polyvinylpyrovidine gel capsule was removed and the microfibrilated cellulose (MFC) powder containing DAO was used directly after dissolving in water, referred to as MFC-DAO. 

**Figure 7:**
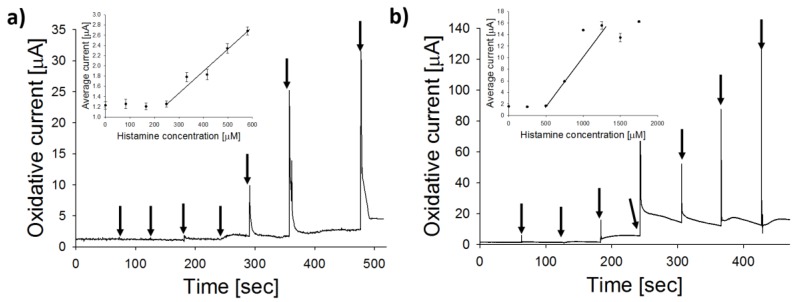
Representative DCPA curves showing histamine oxidation at constant potential of +500 mV. Calibration of (**a**) non-enzymatic electrode (sensitivity = 3.7 µA/mM, LOD = 261 µM); and (**b**) MFC-DAO nanobiosensor (sensitivity = 26.2 µA/mM, LOD = 62.9 µM). Arrows indicate addition of histamine into the electrochemical cell. Insets show the average output and linear operating range.

In order to verify the activity of the locally purchased enzyme, calibration experiments were first performed using copper-coated LSG electrodes in both the absence and presence of MFC-DAO. As shown in Figure 7, the MFC-DAO biosensor shows a 5-fold increase in sensitivity and one order of magnitude decrease in LOD towards histamine, relative to the metallized LSG with no enzyme. This result confirms the catalytic activity of DAO for oxidation of BA, using a locally sourced lyophilized DAO powder. Next, we challenged both biosensors by determining operating characteristics (sensitivity, selectivity, response time, LOD) for various BA in buffer. Subsequently, we compared CNC-DAO and MFC-DAO electrodes for histamine biosensing.

**Figure 8:**
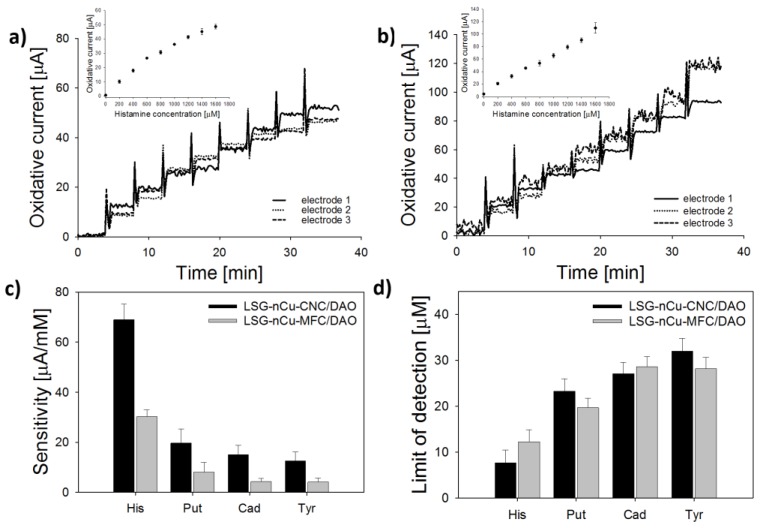
BA calibration for nCu-LSG biosensors prepared with analytical grade materials (LSG-nCu-CNC/DAO) or locally sourced materials (LSC-nCu-MFC/DAO). Representative DCPA time series during stepwise addition of histamine (His) for (**a**) DAO-CNC and (**b**) MFC-DAO biosensors. Average (**c**) sensitivity and (**d**) limit of detection toward various BA. Error bars represent the standard deviation of the arithmetic mean (*n* = 3 sensors).

Representative DCPA time series during stepwise addition of histamine are shown in Figure 8a,b, for each protein type on copper-coated LSG. The histamine sensitivity for analytical grade enzyme, or DAO-CNC (68.7 ± 5.9 µA/mM), was twice as high as sensors prepared with locally sourced enzyme, or MFC-DAO (30.0 ± 1.9 µA/mM), but response times (4 ± 3 s) and linear range (50 µM to 1.6 mM) were similar. Sensitivity toward histamine, putrescine, cadaverine, and tyrosine for each nanobiosensor is shown in Figure 8c. While DAO catalyzes oxidation of different amino acids that may be present in the food matrix, sensors developed here were most selective toward histamine, followed by putrescine, and cadaverine/tyrosine. Histamine is known to be the most common monoamine, and putrescine the most common polyamine, found in food samples, with some exceptions [47]. Cadaverine and tyrosine, while also found in some food samples, typically make up only a small fraction of the total BA [11,18]. The LOD for each biosensor followed the same trend, with the lowest values obtained for histamine, followed by putrescine and cadaverine/tyrosine (Figure 8d). The average histamine LOD for CNC-DAO biosensors (7.7 ± 2.8 µM) was significantly lower than MFC-DAO (12.3 ± 2.6 µM) (*p* = 0.028, α = 0.05), although the LOD for other BA was not significantly different for the two biosensors (*p* < 0.001, α = 0.05). These results clearly show that the LSG-Cu-MFC-DAO biosensors developed with locally sourced materials are a viable tool for rapid screening of BA. In the next section, biosensors were used in tests of fermented fish samples.

### 3.5. Analysis of Fish Samples

**Figure 9:**
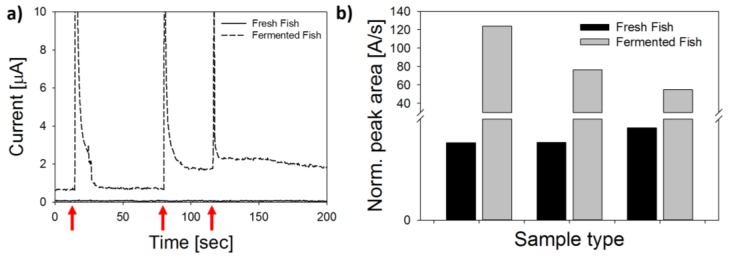
Proof of concept test with BA biosensor fabricated with locally sourced materials. (**a**) Amperometric curves show the electrical response after 3 successive injections of supernatant from non-fermented and fermented fish paste into the electrochemical cell polarized at +500 mV; (**b**) normalized peak area used to calculate total BA content from the 3 successive injections of supernatant from non-fermented and fermented fish paste.

To challenge the LSG biosensors for analysis of food samples, a fish paste was tested before and after solid-state fermentation with lactic acid bacteria. As shown in Figure 9a, an injection of supernatant from the non-fermented sample (noted as “fresh fish”) into the electrochemical cell did not cause an appreciable change in current (less than 1% change in current). Thus, concentration levels were below the LOD of the biosensor (i.e., BA < 2.2 mg-histamine/kg-fish paste). Conversely, significant spikes in oxidative current (≈10 µA) were measured after each successive addition of supernatant from fermented sample into the electrochemical cell. The normalized peak area for fermented and fresh samples is shown in Figure 9b. Raw data for normalized peak area are shown in Supplemental Figure S4. Using normalized peak area, the calculated levels in the fermented sample were 19.24 ± 8.21 mg histamine/kg fish paste (see Supplemental Figure S4 for details).

## 4. Discussion

Biosensors were fabricated via LSG using a low-cost apparatus and locally sourced materials, and compared to electrodes fabricated with analytical grade chemicals. Material analysis of LSG showed a stitched morphology, with an average LSG thickness of approximately 60 µm. The type of copper used for metallization significantly affected the morphology and electrochemical properties of the nanoscale structure, which is not surprising. The mesotube structures formed with locally sourced copper material (from an agricultural store in Cali, Colombia) contained copper/iron alloys, as well as potassium/chloride crystals. The electrokinetics of these microscale mesotube alloys were half as effective as the nanourchin structures with regards to ESA and k^0^. Furthermore, the ∆E_p_ values indicate that the LSG metallized with locally sourced copper is relatively slow. However, when biofunctionalized with DAO (whether analytical grade or locally sourced), the biosensors were selective for BA, with preferential oxidation of histamine over other BA, and both biosensors had a similar response time (≈4–7 s).

Table 1 provides a summary of the data in Figure 2, Figure 3, Figure 4, Figure 5 and Figure 6. Taken together, these data indicate that the amorphous copper structures formed from the locally sourced material are conductive, although the chemical composition and relatively large feature size limit the electrochemical performance somewhat when compared to the biosensors prepared from analytical grade materials. Metallization with analytical grade CuSO_4_ produced high surface area urchin- and crystalline-like copper nanostructures (nCu), while use of locally sourced copper produced relatively low surface area structures that were significantly larger. Peak current and ESA were higher for the urchin-like nanostructures than the mesotube structures, which is expected based on the high surface area of the nanourchin morphology. Furthermore, pulsed electrodeposition with analytical grade CuSO_4_ did not coat the entire graphitized surface, leaving many edge/plane structures of the graphitized material exposed, which is known to significantly enhance chemical reactivity [39,40]. On the other hand, metallization with the locally sourced copper coated the surface with a homogenous amorphous material, likely a result of the surfactants and other trace metals in the solution. This resulted in formation of microscale metal alloys with few exposed edge/plane graphitic structures. Although use of the locally sourced copper material produced a less efficient material, the ESA and k^0^ indicate that the material is viable for use as a sensor (discussed in the following section).

Table 2 summarizes the performance characteristics of each sensor prepared in this work, as well as other similar carbon-metal hybrid nanobiosensors in the recent literature. The CNC-DAO/nCu/LSG sensors (analytical grade) had a sensitivity that was higher than all the recent devices in Table 2, with the exception of the nanoplatinum/DAO/chitosan composite reported by Apetrei and Apetrei [48]. The LOD of the device by Apetrei was significantly lower than the LSG biosensors, but the response time was not reported. While platinum is known to be an excellent nanomaterial for highly efficient carbon-metal hybrid materials [38,49], these materials are expensive, and thus are not applicable in many resource-limited environments. The polymer-nanoceria hybrid by Gumpu et al. [50] showed excellent sensitivity toward histamine, but the LOD was relatively high. The electropolymer (PANI) used in the study by Gumpu et al. [50] is often difficult to obtain in resource-limited environments, and can be cost prohibitive if large numbers of samples must be screened [33]. The DAO/Cu/LSG (locally sourced) biosensor had a sensitivity, which was lower than, but comparable to, many of the devices shown in Table 2. While some devices [48,50] demonstrated enhanced performance over the DAO/Cu/LSG biosensor, these sensors use expensive nanocatalyst metals, and/or cumbersome fabrication methods, which require extensive training and are unlikely to be adopted in resource-limited environments. When using low-cost biosensors such as paper-based devices [23] or the sensors shown here, rapid screening for on-site determination of potentially contaminated food products should be the end goal. The reproducibility of paper/plastic biosensors is relatively low when compared to silicon-based technologies. Also, it must be noted that direct sample analysis (i.e., without pre-treatment steps) always produces interference for any sensor, due to the high chemical complexity in food matrixes. However, the ability to fabricate the sensor platform on-site and produce working biosensors using local materials allows sensor technologies to penetrate applications which were once inaccessible.

Based on the performance characteristics shown in Table 2, biosensors prepared with locally sourced materials are able to screen fermented fish samples in the allowable range (100–200 mg histamine/kg sample) suggested by most regulatory agencies [15,17]. While standard analytical instruments such as thin layer chromatography, high-performance liquid chromatography, gas chromatography, and capillary electrophoresis are highly accurate across this working range, these methods are expensive and time consuming, requiring several sample pretreatments and highly skilled operators. Although biosensors have been developed for screening BA (as shown in Table 2), many of the devices are cost prohibitive or difficult to fabricate, particularly for applications in developing countries or economically challenged rural areas, where access to a laboratory is limited.

The cost for screening BA in food samples in Cali, Colombia is shown in the Supplemental Table S1. Local companies were contacted to determine analytical costs per food sample, including ELISA and HPLC (the two most common methods). For estimating the cost of the enzymatic biosensor developed herein, local costs of the materials and equipment required for the fabrication of 17,942 biosensors per year (which is the number of devices that can be fabricated by one single person working 40 h/week) were taken into account. In terms of equipment, calculations assumed 10 years for return on investment with 10% annual depreciation. Other costs such as personnel, utilities, maintenance, rent, etc., were not considered in the calculation. Profit was also not included in the calculation. The estimated cost was $0.42 dollars per device. Overall, for a low throughput screening of samples, enzymatic biosensors are the most economical tool (costing less than $1 USD), and sample analysis can be conducted in approximately one hour. In addition to these advantages (which are common for biosensors), the total manufacture time for producing a biosensor was approximately 1.5 h, including laser-scribing, electroplating, Ag/AgCl deposition, passivation, and biofunctionalization; the fabrication process did not require the use of exotic or expensive equipment. Together, this represents an important new tool in food safety analysis that can be reproduced in any lab. The biosensors here represent a component of open source appropriate technology (OSAT) [56], that can be used by local community members for monitoring food safety.

## 5. Conclusions

Global demand for food, water, energy and public health infrastructure are increasing [57], and sensor-based solutions aiming to solve specific problems related to world health should be sustainable and reproducible [58]. The convergence of nanotechnology and public health knowledge requires technology transfer in diverse scenarios—ranging from developing regions to urban centers [59]. Open source appropriate technologies, such as the biosensors developed here, are tools that can provide useful data regarding the safety and quality of food to consumers who lack access to chemical testing laboratories. Open source technologies must not only consider recent technological innovations and public health scenarios, but also how users can recreate the technology using local resources, thus facilitating long-term adoption with limited outside assistance. This work is the first step toward convergence of LSG biosensors with rural communities for improving public health while simultaneously limiting food waste. The results show that LSG biosensors can be fabricated in economically challenged areas by using locally available materials sourced from supermarkets and agricultural stores. The sensors provide rapid screening of food toxins such as biogenic amines, for a fabrication cost of less than $1 USD. Although not discussed in detail, the convergence of mobile phone-based acquisition hardware [60,61,62] and ad hoc data analysis with machine learning [63] represent a complete solution for food safety and security problems in developing regions using the biosensors developed here. As these technologies progressively converge, the portfolio of planetary health sensors will grow, and data-driven decision making can expand to difficult-to-access regions, where it is needed most.

## Figures and Tables

**Table 1:** Summary of material characterization and electrochemical parameters for LSG with two different sources of copper used for metallization.

Material	Feature Size (Morphology)	Material Identified in Features (keV)	^1^ Peak Redox Current (µA)	^2^ ESA (cm^2^)	^3^ HET Constant (-)
LSG	NA	C (0.28 keV) O (0.54 keV)	≈100	0.34 ± 0.05	0.0030 ± 0.0011
^4^ LSG + nCU (CuSo_4_ source)	25 to 300 nm (urchin, crystallite)	Cu (0.92 keV) Cu (8.1 keV)	≈500	0.46 ± 0.06	0.0038 ± 0.0017
^4^ LSG + nCU (local source)	500 to 1000 nm (rod, mesotube)	Cu (0.92 keV) Cu (8.1 keV) K (3.3 keV) Cl (2.6 keV) Fe (6.97 keV)	≈280	0.50 ± 0.21	0.0033 ± 0.0020

^1^ Data for scan rate of 100 mV/s; ^2^ ESA = electroactive surface area; ^3^ HET = heterogenous electron transfer constant; ^4^ data shown for 60 s of electrodeposition.

**Table 2:** Average performance characteristics of enzymatic biosensors for biogenic amines determination.

Sensor Conformation	Food Sample (BA)	Sensitivity (µA/mM)	LOD (µM)	t_95_ (s)	Reference
DAO/Pt-NP/Graphene/Chi/SPE	fish (His)	63.1	0.02	NR	Apetrei [48]
DAO-HRP/polysulfone/CNT/ ferrocene/SPE	fish (His)	19	0.17	20	Pérez [51]
DAO/photoHEMA/SPE	prawn (His)	0.62	5.8	50	Keow [52]
DAO/CeO_2_-PANI/GCE	prawn (His)	51.47	48.7	1	Gumpu [50]
DAO/Nafion/MnO_2_/SPE	chicken (His/Tyr)	5.95	3.0	NR	Telsnig [53]
DAO-HRP/SPE	fish (His)	17.66	0.18	NR	Alonso-Lomillo [54]
HMD/TTF/SPCE	octopus (His/Put)	10.2	8.1	NR	Henao-Escobar [55]
LSG-nCu-CNC/DAO (analytical grade materials)	fish (His)	58.7 ± 5.9	7.7 ± 2.8	4.1 ± 1.7	This work
LSG-Cu-MFC/DAO (locally sourced materials)	fish (His)	23.3 ± 1.9	11.6 ± 2.6	7.3 ± 2.5	This work

NR: not reported in the manuscript; His = Histamine.

## Supplementary Materials

The following are available online at http://www.mdpi.com/2079-6374/8/2/42/s1. Figure S1: Overview of LSG electrode fabrication Figure S1. Fabrication process for LSG sensor platform. (**a**) Photograph of polyimide tape deposited on emulsion side of photopaper; (**b**) photograph of pulsed UV laser inscribing system; (**c**) three electrode LSG sensor on polyimide before and after functionalization. Image on the right shows passivation of connecting wires with lacquer polish (red), painting of reference electrode with Ag/AgCl paint, and nCu deposition on the working electrode. The diameter of the working electrode is 4 mm. Figure S2: Profile of LSG electrodes. (**a**) Low resolution SEM image showing polyimide + LSG (dark grey material) and cellulose backing (white material); (**b**) high resolution SEM image of carbonized LSG. Figure S3: XPS spectra of LSG, and metallized LSG with two different copper sources. Peaks associated with C1s, (carbon) N1s (nitrogen), O1s (oxygen) and Cu2p (copper) are highlighted. Figure S4: Raw amperometric data indicating oxidation of BA after addition of (**a**) fermented fish and (**b**) fresh (non-fermented) fish. Replicate injections of sample are overlayed in each image and the equation for calculating normalized peak area shown. After converting to BA concentration via calibration curves, the total BA (in μg histamine/g fish paste) was calculated for each sample. Table S1: Cost summary of methods for the determination of histamine available in Colombia. Quotes for RIDASCREEN and HPLC were obtained from standardized laboratories in Colombia. The estimated cost of the test using the enzymatic biosensor only accounts for the device fabrication.
