# Supplementary material for: Laser Scribed Graphene Biosensor for Detection of Biogenic Amines in Food Samples Using Locally Sourced Materials
Source: Biosensors (Basel). 2018 Apr 24;8(2):42. doi: 10.3390/bios8020042 (PMC6023090; doi:10.3390/bios8020042)
Supplement: Supplementary file 1 [file biosensors-08-00042-s001.pdf]

biosensors

The prioritized criteria for materials selection is low cost and high availability, where compounds such as the copper-containing fertilizer solution were intentionally generic and common. Materials such as polyimide tape (also known as Kapton tape) are generally available in almost any electronics store. To verify the widespread availability of these two key materials, the authors have personally verified local availability in the USA, Canada, Mexico, Colombia, China, Brazil, Malaysia, Indonesia, India, and the Bahamas (for example). Although, there are several brands and qualities, the basic composition is the same for these two key materials.

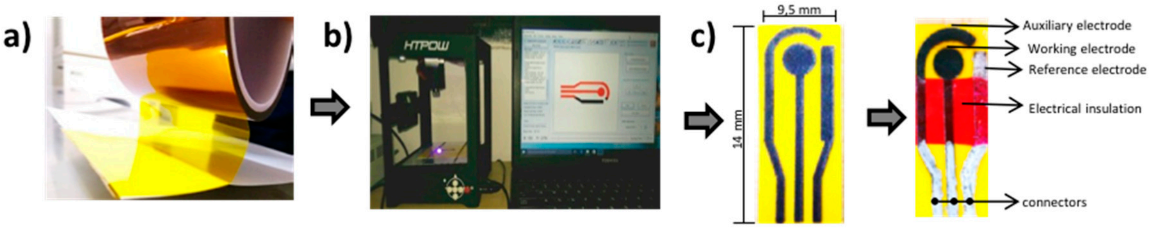

Figure S1. Overview of LSG electrode fabrication Figure S1. Fabrication process for LSG sensor platform. (**a**) Photograph of polyimide tape deposited on emulsion side of photopaper; (**b**) photograph of pulsed UV laser inscribing system; (**c**) three electrode LSG sensor on polyimide before and after functionalization. Image on the right shows passivation of connecting wires with lacquer polish (red), painting of reference electrode with Ag/AgCl paint, and nCu deposition on the working electrode. The diameter of the working electrode is 4 mm.

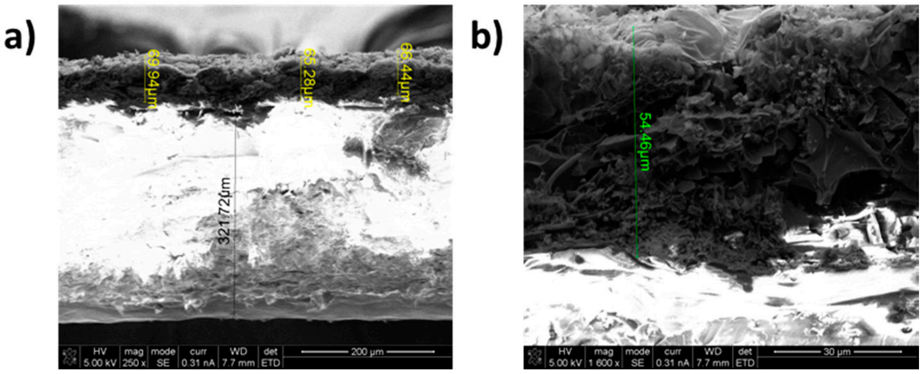

Figure S2. Profile of LSG electrodes. (**a**) Low resolution SEM image showing polyimide + LSG (dark grey material) and cellulose backing (white material).; (**b**) high resolution SEM image of carbonized LSG.

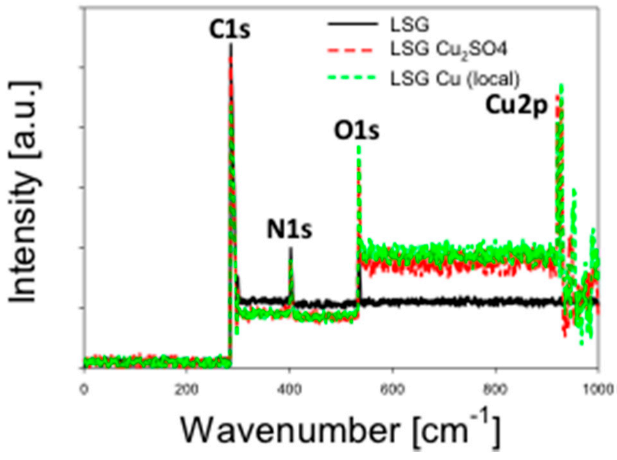

Figure S3. XPS spectra of LSG, and metallized LSG with two different copper sources. Peaks associated with C1s, (carbon) N1s (nitrogen), O1s (oxygen) and Cu2p (copper) are highlighted.

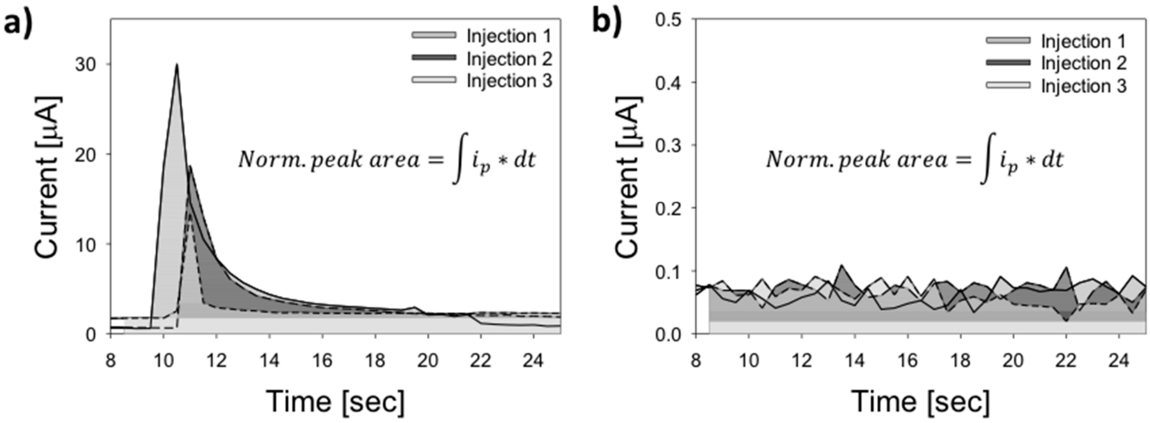

Figure S4. Raw amperometric data indicating oxidation of BA after addition of (**a**) fermented fish and (**b**) fresh (non-fermented) fish. Replicate injections of sample are overlayed in each image and the equation for calculating normalized peak area shown. After converting to BA concentration via calibration curves, the total BA (in μg histamine/g fish paste) was calculated for each sample.

Table S1. Cost summary of methods for the determination of histamine available in Colombia. Quotes for RIDASCREEN and HPLC were obtained from standardized laboratories in Colombia. The estimated cost of the test using the enzymatic biosensor only accounts for the device fabrication.

Technology	Description	No. Samples Screened per Test	Analysis Time	Cost per Test [COP] (USD)
RIDASCREEN Histamine	Direct ELISA for quantitative determination of histamine in food samples	96	90 min	[$2082065] ($750)
RIDASCREEN Histamine (Enzymatic)	Sandwich ELISA for quantitative determination of histamine in fresh fish, canned fish, fish flour, cheese and milk	96	30 min	[$2279455] ($820)
HPLC*	Chromatographic method for quantitative determination of histamine in fish	1	8 days	[$224100] ($80)
Enzymatic Biosensor * (this work)	LSG-Cu-MFC/DAO (locally sourced materials)	1	60 min	[$1181] ($0.42)

COP—Colombian Peso; USD—US Dollar. * Tests are done in triplicate.
